# Study on Leakage Effect Factors of Two-Stroke Micro Free Piston Swing Engine

**DOI:** 10.3390/mi13081314

**Published:** 2022-08-14

**Authors:** Shujing Miao, Haiyang Liu, Haitang Cen, Jiang Liu, Huaqiang Li, Gang Xu

**Affiliations:** School of Mechanical Engineering, Inner Mongolia University of Technology, Hohhot 010051, China

**Keywords:** seal gap, micro free piston swing engine, mass leakage, size factor

## Abstract

The two-stroke micro free piston swing engine (MFPSE) is a portable power device. Its seal performance plays an important role in the dynamic properties and efficiency of microengines. The present work established the leakage model of the two-stroke micro free piston swing engine by utilizing the compressible flow Reynolds Navier–Stokes equation. The obtained nondimensional mass leakage was related to the seal gap height, seal inlet pressure, size factor and compression ratio. Simulation investigated how the different seal gap heights and size factors affected the pressure, temperature and mass leakage of micro engines. The results showed that when the seal gap height of the combustion chamber increased, the maximum pressure and cycle power declined, obviously. However, the maximum temperature was scarcely affected. The mass leakage was not greatly impacted when the compression ratio was less than 5. However, the mass leakage dramatically increased when the seal gap was more than 10 μm or the size factor was less than 0.4. The investigation revealed these mass leakage effect factors and provided a guide for the seal and structure design of the two-stroke micro free piston swing engine.

## 1. Introduction

In recent years, micro electro-mechanic systems (MEMS) productions such as microrobots, micromotors, microsensors and micro-aerial vehicles, have been widely applied in the aerospace, military, and medical fields. As the dominant power supply for those portable devices, batteries have been unable meet the small size, low weight and long duration requirements. Thus, developing power supply systems to meet these requirements are required [1]. However, microengines utilizing liquid hydrocarbon fuel with high energy density have proven able to meet requirements, and have become a major research hotspot [2,3]. A micro gas turbine engine was first proposed by Epstein for use as MEMS power supply [4]. Subsequently, micro rotary engines [5], micro free piston engines [6] and micro internal combustion swing engines [7] were proposed and developed. Moreover, compared with other microengines, the micro free piston swing engine has the advantages of simple structure, large space utilization, and low swing frequency. As such, it is a great potential microengine for providing the necessary power for MEMS productions. However, with the size reduction of a microengine, its combustion, heat transfer and mass leakage will obviously differ from those of conventional engines; that has restricted the development of a micro internal combustion engine.

At present, many research groups have developed combustion processes for microengines. Lee believed that efficiency in small-scale combustors and the prescribed minimum size of the combustor were two main issues [8]. Ghobad showed that the thermal cycle of a microcombustor had an important influence on the flame stability and performances [9]. Ren investigated the size influence of microcombustion and the ignition conditions of microengines [10]. Hua’s study determined that the influences of residence time, chemical reaction time, thermal quenching and the size of the combustion chamber were important factors for microengine combustors [11]. Aichlmayr explored different diameters and lengths effect of small-scale free piston homogeneous charge compression ignition in combustion experiments [12]. The above studies showed that the critical factors of microengine combustion were the combustion process and combustor size.

In addition to microengine combustion, heat transfer and leakage are additional concerns [13,14]. Moreover, the leakage is a special key factor. Due to the presence of leakage, the pressure of combustion/scavenge chamber drops. As a result, the thermal efficiency of a microengine obviously declines—or the engine could fail to work correctly at all. For micro homogeneous charge compression ignition engines, mass leakage and thermal efficiency were impacted by the cylinder-piston gap, engine speed and compression ratio [15,16]. For micro rotary engines, the combustion chamber size and cycle efficiency were related to the engine speed, apexes gap, and compression ratio [17]. For a four-stroke micro internal combustion swing engine (MICSE), Gu found that the leakage dramatically dropped the operation conditions of the prototype [18]. Zhang proposed and studied the two-stroke micro free piston swing engine [19]. However, when the body-center swing gap was small, the center swing would be glued to the body after working some time. On the other hand, when the seal gap was large, the two-stroke MFPSE was unable to operate properly. A proper seal gap is a key factor affecting leakage in an MFPSE. The above studies clearly showed that microengine leakage was one of the main problems restraining its development.

In order to explore the leakage problem in micro swing engines, Sun presented a groove seal structure for a micro internal combustion swing engine and analyzed its flow characteristics. It was found that the seal performance was affected by the width and depth of the groove [20]. Shi showed that the thermal efficiency of a micro internal combustion swing engine was affected by various factors, including the leakage, heat transfer, friction, and size factor [21]. Zhou showed that, when the seal gap was less than 3 μm, the mass leakage of a three-arm micro swing engine was not strongly affected, by considering the wall slid of flow regime and rarefaction effect of the Knudsen number. Zhou also studied the effects of thermal efficiency on the microengine size factor [22]. Du established the leakage model, which was in the slip flow range but close to the continuous flow, and discovered that mass leakage was the main contributor to energy loss in a micro swing engine through experimentation and numerical simulation [23]. The above studies mainly investigated leakage in four-stroke micro internal combustion swing engines. However, the working process and structure of a two-stroke micro free piston swing engine differ from those of a four-stroke MICSE, despite their similar appearance [24]. Moreover, for the two-stroke MFPSE, the influence of leakage has not been studied sufficiently; it needs to be investigated further.

In this paper, for the two-stroke MFPSE, the mass leakage model was established by considering the compressible fluid of seal controlled volume, and the relationships between nondimensional mass leakage and effect factors were theoretically explored. Finally, numerical simulation was used to investigate how the seal gap height, size factor, and compression ratio affected the mass leakage. This study provided a guide that could be used to improve body-center swing seal performance and aid in miniaturization of the structure of the MFPSE.

## 2. Structure and Working Principle of MFPSE

### 2.1. Structure Description

**Figure 1:**
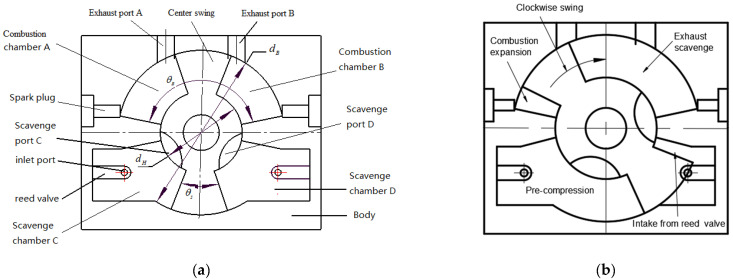
Schematic diagram and work principle of MFPSE; (**a**) schematic diagram; (**b**) work principle.

The two-stroke micro free piston swing engine is a micro power device converting the chemical energy of the fuel into mechanical energy by swinging the center swing in a fixed cavity. It has variable compression ratio and its power output is in the form of swing. Figure 1a and Table 1 show its structure schematic and parameters of MFPSE, respectively. The center swing divides the upper and lower cavities of the body into combustion chambers A and B and scavenging chambers C and D. The compressible fresh charge in the scavenge chamber enters the combustion chambers through scavenge port C or D and then undergoes compression and combustion.

### 2.2. Working Principle

The working principle of the two-stroke micro free piston swing engine is shown in Figure 1b. We assumed that combustion chamber A was in the initial position (left limit) of the combustion process, and a closed area was formed by the body, the center swing, and the cylinder head. When the spark plug ignited the mixture in combustion chamber A, the mixture pushed the center swing to the right limit of combustion chamber A. At this time, combustion chamber B was in the initial state of the compression process, and scavenging port D and exhaust port B were in the open state. The scavenging process began with a fresh charge and swept out the existing gas in combustion chamber B. The fresh charge in scavenge chamber C was in the initial state of the precompression process. The pressure of the fresh charge increased with the movement of the center swing. Meanwhile, scavenge chamber D was in the initial position of the intake process. When the center swing moved clockwise to the right limit position of combustion chamber A, combustion chamber B was in the initial position of the combustion process. Then, combustion chamber B and A repeated the stroke cycles by turn. This was the working principle of the two-stroke micro MFPSE.

## 3. Leakage Model Established

For the two-stroke MFPSE, the mixture in the combustion/scavenge chambers was sealed by the body, the center swing and the cylinder head, so the seal gap height and inlet pressure exerted powerful effects on the mass leakage of combustion/scavenge chambers. At this time, the pressure and temperature of the seal inlet were those of combustion/scavenge chambers.

### 3.1. Pressure and Temperature of Combustion/Scavenge Chamber

In order to obtain the pressure and temperature of the combustion/scavenge chambers, the first law of thermodynamics for the ideal gas law and an open system were applied. The pressure and temperature derivatives of the combustion/scavenge chambers are defined as follows [25]:

(1) $${\dot{T}}_{c}=\frac{RT_{c}}{C_{v}}\left[{\dot{x}}_{b}(\frac{R_{b}-R_{u}}{R}+\frac{h_{u}-h_{b}}{RT_{c}})+\frac{\dot{m}}{m}-\frac{\dot{V}}{V}\right]+\frac{1}{mC_{v}}\left[\sum_{i}{\dot{m}}_{i}h_{i}-{\dot{Q}}_{w}\right]$$

(2) $${\dot{P}}_{c}=P_{c}\left[{\dot{x}}_{b}\frac{R_{b}-R_{u}}{R}+\frac{{\dot{T}}_{c}}{T_{c}}+\frac{\dot{m}}{m}-\frac{\dot{V}}{V}\right]$$

where $P_{c}$ and $T_{c}$ are the pressure and the mean mixture temperature inside controlled volume, respectively; R represents the controlled volume’s mixture gas constant; the subscripts b and u denote the burned and unburned gas, respectively; $x_{b}$ is the burned mass fraction; $C_{v}$ is the specific heat under constant volume; m is the total mass inside the controlled volume; V represents the instantaneous controlled volume; $m_{i}$ and $h_{i}$ are the mass and the specific enthalpy flow into/out of the controlled volume respectively; the subscript i denotes the species in the mixture and fuel; ${\dot{Q}}_{w}$ is the heat transfer loss cross the system boundary.

For the microengine, the convective heat transfer loss and the heat loss in unit time are expressed as [26]:

(3) $$\frac{dQ_{w}}{dt}=h_{c}A_{s}(T_{c}-T_{w})$$

In Equation (3), the mean heat transfer coefficient $h_{c}$ is expressed by Hohenberg [26]:

(4) $$h_{c}=130V^{-0.06}{\left(\frac{P_{c}}{10^{5}}\right)}^{0.8}T_{c}^{-0.4}{\left(U_{0}+1.4\right)}^{0.8}$$

In Equations (3) and (4), $A_{s}$ is the surface area of the corresponding combustion/scavenge chambers, $T_{w}$ is the mean wall temperature, $U_{0}$ is the mean velocity of center swing.

### 3.2. Leakage Model

**Figure 2:**
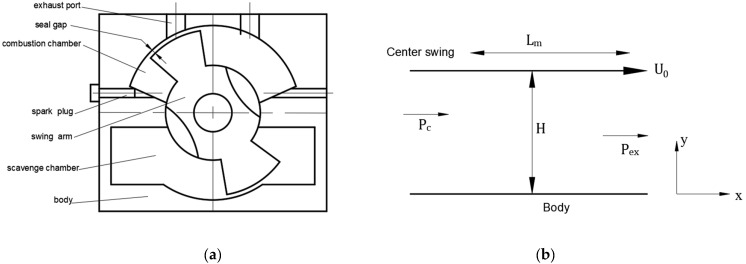
Leakage model of MFPSE; (**a**) physical model; (**b**) mathematical model.

For the two-stroke MFPSE, Figure 2a shows the physical leakage model. The mixture of the combustion chamber entered the exhaust port through the body-center swing seal gap. Then, the mixture flow of seal gap was thought as the clearance flow, because the fluid behavior can be represented as continuity flow condition if the passages of gas flow were more than 1 μm [27]. Hence, the Poiseuille–Couette continuity equations can be used for the gap seal flow of MFPSE. Figure 2b indicates the mathematical leakage model of MFPSE. We assumed the gap seal flow as follows:

(1) The gap height between the body and the center swing was significantly smaller than the center swing radius, and thus the gap seal was modeled as a moving semi-infinite plane. In the same way, ∂P/∂y≈0.
(2) The quasi-static laminar, one-dimensional, and compressible flow was through the leakage gap.
(3) Flow was viscous, taking the mixture as the working fluid.
(4) We assumed no slip on the center swing or body walls.

According to the assumptions above, the continuity equation of seal flow can be obtained:

(5) $$\frac{1}{\mu }\frac{\partial P}{\partial x}=\frac{d^{2}u}{dy^{2}}$$

In Equation (5), the velocity profile inside seal gap is expressed by utilizing the variable separation technique and considering the two terminal conditions (y=0 with u=0, y=H with $u=U_{0}$):

(6) $$u=\frac{1}{2\mu }\frac{\partial P}{\partial x}(y^{2}-yH)+U_{0}\frac{y}{H}$$

In Equations (5) and (6), x is the coordinate along the body length, y is the coordinate along the seal gap height, H is the seal gap height between the center swing and the body, u and P are x velocity component and the pressure inside the leakage path, separately, μ is the dynamic viscosity coefficient, $U_{0}$ is the mean velocity of center swing.

The mean velocity of the seal flow $u_{m}$ is as follows:

(7) $$u_{m}=-\frac{1}{12\mu }\frac{\partial P}{\partial x}+\frac{U_{0}}{2}$$

For Equation (7) right side, the first part is the pressure-driven flow through the seal gap. The second part is the volume flow rate, because of the moving center swing. The Couette flow rate was smaller than the Poiseuille flow rate. Therefore, the Couette flow was negligible [17]. The mass leakage flow rate of gap seal for MFPSE is expressed:

(8) $$\dot{m}=\rho Au_{m}=\rho HB(-\frac{1}{12\mu }\frac{\partial P}{\partial x})$$

where ρ is the flow density, A is the cross area of seal flow, and B is the seal flow width.

Since the high-pressure gradient through the seal gap is a typical feature of mass leakage flow, the compressible gas inside the sealed volume was considered. The compressible flow Reynolds Navier–Stokes equation was used for the leakage of MFPSE:

(9) $$\frac{\partial }{\partial x}(\frac{\rho H^{3}}{\mu }\frac{\partial P}{\partial x})+\frac{\partial }{\partial z}(\frac{\rho H^{3}}{\mu }\frac{\partial P}{\partial z})=6\frac{\partial }{\partial x}(\rho HU_{0})+6\frac{\partial }{\partial z}(\rho HW)+12\frac{\partial }{\partial t}(\rho H)$$

For Equation (9), we assumed that ∂/∂z=0 (symmetry), and that $\frac{\partial (\rho H)}{\partial t}=0$ (quasi-static process). Meanwhile, due to the negligible Couette flow ($U_{0}=0$), we obtained the simplified N-S equation:

(10) $$\frac{\partial }{\partial x}(\frac{\rho H^{3}}{\mu }\frac{\partial P}{\partial x})=0$$

For the mass leakage of sealed volume, the mixed gas temperature increased with the decrease of the pressure along the leakage path. It was supposed that the flow temperature inside the seal gap increased with the heat derived from Poiseuille flow. Based on the assumption above, we obtained

(11) $$T=\frac{P_{c}-P}{\rho C_{p}}+T_{c}$$

where P and T are the instantaneous pressure and temperature inside the sealed volume respectively, $P_{c}$ and $T_{c}$ are the pressure and the temperature of the seal inlet mixture respectively, and $C_{p}$ is the specific heat. According to the ideal gas equation and Equation (11), we obtained the mathematical expression of density and pressure inside sealed volume:

(12) ρ=aP−b

where $a=\frac{1}{T_{c}R_{c}}+\frac{1}{T_{c}C_{p}}$ and $b=\frac{P_{c}}{T_{c}C_{p}}$.

Substituting Equation (12) into Equation (10), the pressure P in Equation (10) was integrated in the first and the second by using variable separation technique, respectively. It was obtained, as follows:

(13) $$\frac{\partial P}{\partial x}=\frac{C_{1}\mu }{H^{3}}\frac{1}{aP-b}$$

and:

(14) $$\frac{1}{2}aH^{3}P^{2}-bH^{3}P=C_{1}\mu x+C_{2}$$

In this study, due to large pressure gradients through the seal gap, subsonic flow and choke flow were distinguished.

For the subsonic flow regime, considering the boundary conditions, we obtained

(15) $$x=0\text{ and }P=P_{c}\;\frac{1}{2}aH^{3}P_{{}^{C}}^{2}-bH^{3}P_{C}=C_{2}$$

(16) x=Lm and P=Pat     12aH3Pat2−bH3Pat=C1μLm+C2

where $L_{m}$ is the leakage path’s length and is related to the swing angular, and $P_{at}$ is the atmosphere pressure. From there, $C_{1}$ can be solved by substituting Equation (15) into Equation (16). Once $C_{1}$ is given, Equation (13) is used to obtain ∂P/∂x. We obtained

(17) $$\frac{\partial P}{\partial x}=\frac{a(P_{at}^{2}-P_{c}^{2})-2b(P_{at}^{}-P_{c}^{})}{2L_{m}(aP-b)}$$

For the choke-flow regime, the mean velocity of mixture outlet in sealed volume $u_{ex}=\sqrt{\gamma RT_{ex}}$. Based on the ideal gas equation and Equation (14), we obtained

(18) $$\sqrt{\frac{\gamma RT_{c}P_{ex}C_{p}}{P_{ex}(C_{p}+R)-P_{c}R}}=\frac{H^{2}}{12\mu }\frac{a(P_{c}^{2}-P_{ex}^{2})-2b(P_{c}^{}-P_{ex}^{})}{2L_{m}(aP-b)}$$

where the trial and error procedure is used to solve $P_{ex}$ (the critical pressure for the choke flow).

Then the mass leakage flow rate of sub-sonic flow and choke flow were estimated by substituting Equations (17) and (18) into Equation (8), respectively. 

### 3.3. Nondimensional Mass Leakage

For the two-stroke micro free piston swing engine, in order to estimate the mass leakage effect of the combustion/scavenge chamber, the nondimensional mass leakage was used as an effective evaluation method. It is defined as follows [12]:

(19) $$M_{L}=\frac{\int_{0}^{\tau }\dot{m}dt}{m_{0}}=\frac{\int_{0}^{\tau }\rho HBu_{m}Hdt}{m_{0}}$$

where $M_{L}$ is the nondimensional mass leakage, τ is the center swing working time during compression process of the combustion/scavenge chamber, $m_{0}$ is the fresh charge of combustion/scavenge chamber at the beginning of compression process. The initial mass of combustion chamber is expressed as follow:

(20) $$m_{0}=\frac{P_{0}}{R_{0}T_{0}}\frac{d_{B}^{2}-d_{H}^{2}}{8}\pi \theta_{E}B$$

where $P_{0}$, $T_{0}$, $R_{0}$ are the combustion chamber’s initial pressure, initial temperature and initial gas constant during compression process, respectively, $d_{B}$, $d_{H}$ are the outer and the inner diameter of the center swing, respectively, and $\theta_{E}$ is the exhaust angle of the combustion chamber.

In Equation (20), the mass of combustion chambers was related to the diameter of center swing. In order to discuss the different sizes influence of microengine, the nondimensional size factor λ is defined as $\lambda =(d_{B}-d_{H})/d_{H}$.

Substituting Equations (8), (12), (17) and (20) into Equation (19), the combustion chamber’s mass leakage of the two-stroke MFPSE is expressed:

(21) $$M_{L}=K_{L}\frac{P_{in}^{2}}{P_{0}}\frac{H^{2}\ln(\gamma_{V})}{\mu \lambda^{2}(\lambda +2)}$$

where $p_{in}$ is the seal inlet pressure, $\gamma_{V}$ is the compression ratio of the combustion chamber, and $K_{L}$ is the coefficient of the nondimensional mass leakage, as follows:

(22) $$K_{L}=\frac{2}{3\pi \theta_{E}d_{H}^{3}}(\frac{2}{(\gamma +1)\gamma }+\frac{4(\gamma -1)}{(\gamma +1)\gamma }{\left(\frac{2}{\gamma +1}\right)}^{\frac{\gamma }{\gamma -1}}-\frac{2(2\gamma -1)}{(\gamma +1)\gamma }{\left(\frac{2}{\gamma +1}\right)}^{\frac{2\gamma }{\gamma -1}})$$

where γ is the specific heat ratio.

As shown in Equation (21), the mass leakage of the combustion chamber for MFPSE was related to the seal gap height, size factor, compression ratio, mixture dynamic viscosity coefficient, and seal path inlet pressure. Additionally, the mass leakage had a nonlinear relation with the parameters above.

### 3.4. Model Validation

**Figure 3:**
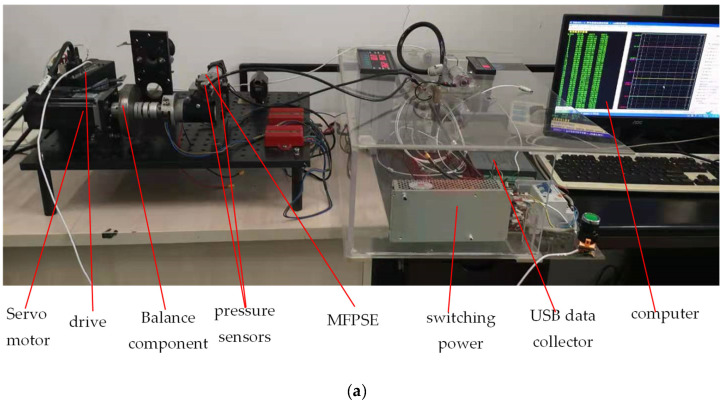

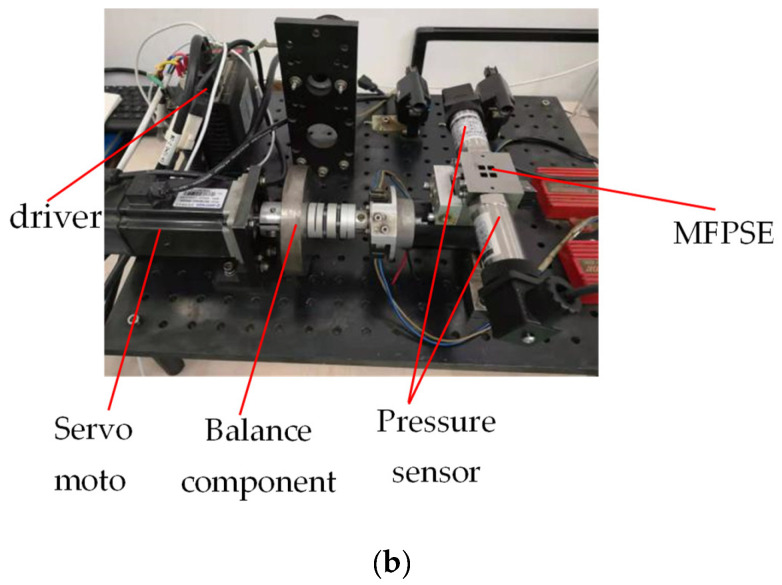
Experimental test workbench for MFPSE: (**a**) whole test workbench; (**b**) mechanical test components of MFPSE.

To validate the mass leakage model based on the compressible flow Navier–Stokes equation, an experimental test workbench was set up. The outer and inner diameters of the center swing were 32 mm and 18 mm, respectively. The body-center swing seal gap was 10 μm. The pressure transducer was STP-TC2R08TG0 (0~1.6MPa) by Sailing Technology. The data collector used was a USB5831 by ART Technology. In order to avoid the expansion of center swing causing the seal gap to change during the combustion process, a servo motor drove the two-stroke MFPSE while the experiment was carried out. The experimental test workbench is shown in Figure 3a. In order to display the mechanical parts of the test workbench, the mechanical test components of Figure 3a are enlarged and shown in Figure 3b.

**Figure 4:**
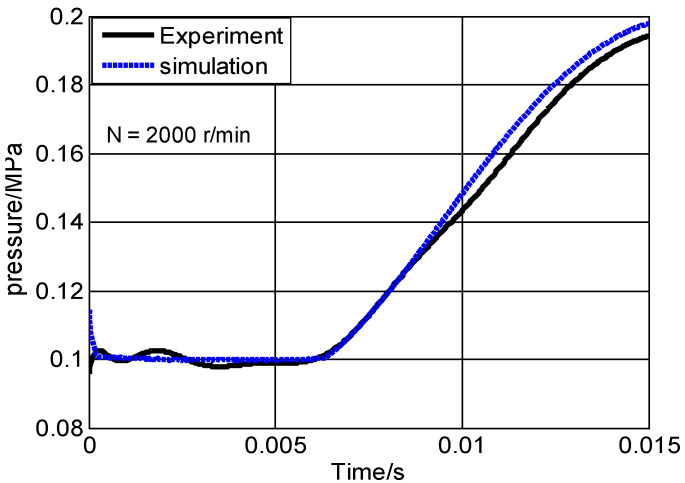
Comparison of experimental and simulation pressure.

For the two-stroke MFPSE, when its structure parameters were constant, the pressure of the combustion chamber was the key factor affecting mass leakage. Figure 4 showed the comparison of experimental and simulation pressure for the combustion chamber during the compression process. The experimental curve was formed by fitting the collection data points. Simulation curve was obtained by the pressure, $P_{c}$, in Equation (2), whereas the mass leakage, $\dot{m}$, was obtained by the leakage model of the compressible flow Reynolds Navier–Stokes equation. In the initial stage of the compression process, the experimental pressure was in good agreement with the simulated pressure. Over time, the simulation pressure was slightly more than the experimental one, but the maximum error as only 1.03% at the endpoint of the compression process. This comparison indicated that the simulation leakage model of MFPSE was both reasonable and valid.

## 4. Results and Discussion

For the two-stroke MFPSE, the seal performance directly affected the mass leakage. Then, the leakage was able to cause decreases in engine power and economy of performance. In this paper, we explored the different microengine size factors and seal gap heights, and how they influenced the combustion chamber temperature, pressure, and mass leakage using numerical simulation. In this section, numerical simulations were based on the structure and the parameters of MFPSE, as shown in Figure 1 and Table 1.

### 4.1. The Effects of Pressure at Different Seal Gap Heights and Size Factors

**Figure 5:**
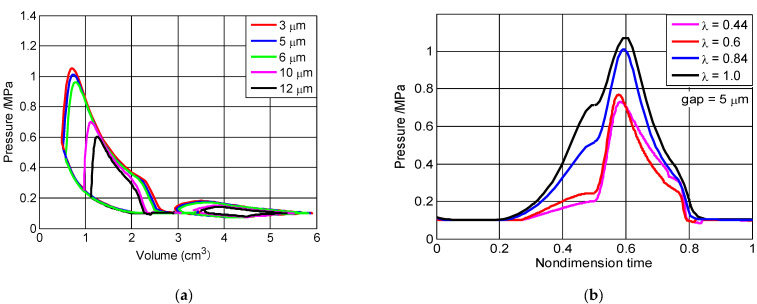
The influence of pressure (**a**) with volume of combustion/scavenge chambers for different seal gap heights; and (**b**) with the nondimensional time cycle for different size factors.

As shown in Figure 5a, when the size factor was 0.84, the body-center swing seal gap varied from 3 μm to 12 μm. The maximum pressure and the cycle power declined by 42% and 40%, respectively. Under the same size factor, the maximum pressure and the cycle power declined with the increase of the seal gap. However, when the gap varied from 3 μm to 5 μm, the cycle power was almost constant, although the maximum pressure slightly declined. Therefore, the 5 μm gap was chosen as a numerical simulation. Figure 5b shows how the size factors affected the pressure of combustion chambers during the nondimensional cycle, as the 5 μm gap. When the size factor varied from 1.0 to 0.44, the maximum pressure of the combustion chambers declined by 28% and were slightly offset. This occurred primarily because the mass leakage increased—and the actual engine speed decreased—with the reduction of size factors.

### 4.2. The Influence of Temperature for Different Size Factors

**Figure 6:**
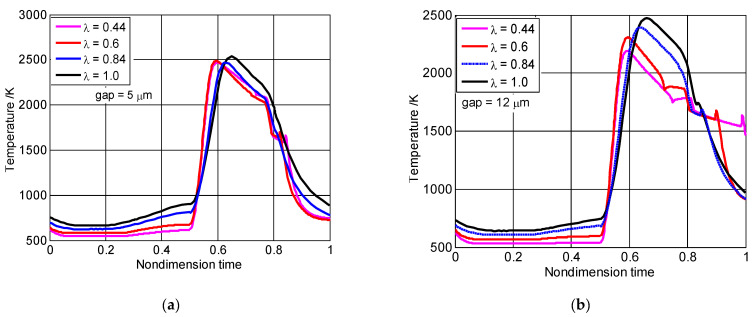
The influence of temperature on the nondimensional time cycle for different size factors: (**a**) the 5 μm seal gap, (**b**) the 12 μm seal gap.

Figure 6 shows how the size factors affected the temperature of the combustion chamber during the nondimensional cycle when the seal gaps were 5 μm and 12 μm, respectively. During the compression process, the temperature of the combustion chamber declined with the reduction of size factors. As shown in Figure 6a, for the 5 μm seal gap height, the combustion chamber’s maximum temperature was almost 2500 K, although the size factors were different. As shown in Figure 6b, for the 12 μm seal gap height, when the size factor varied from 1.0 to 0.44, the maximum temperature of the combustion chambers decreased by 11%, and the final temperature of the combustion chambers for the 0.44 size factor was higher than that of the three other size factors. This was mainly because the relative mass leakage rapidly increased and the compression ratio reduced. Hence, it was clear that, for the small seal gap, the maximum temperature was almost unaffected even when the size factors are different. However, for the large seal gap, the maximum temperature obviously declined with reductions in size factor.

### 4.3. The Effect of Mass Loss for Different Size Factors

**Figure 7:**
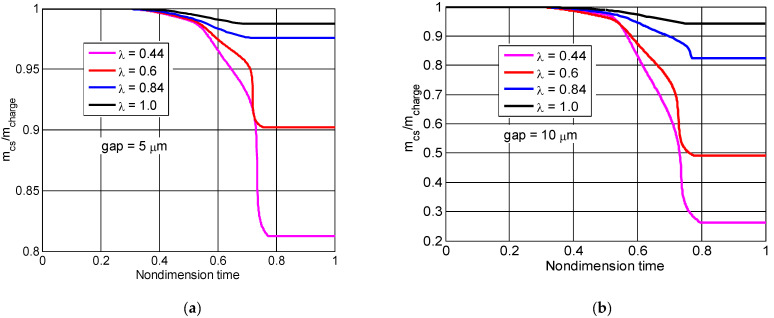
The effect of mass percentage in the nondimensional time cycle for different size factors (**a**) the 5 μm seal gap, (**b**) the 10 μm seal gap.

Figure 7 shows how size factors affected the mass percentage of the combustion chambers during the nondimensional cycle when the gaps were 5 μm and 10 μm, respectively. $m_{\mathrm{charge}}$ and $m_{cs}$ represent the charge and the instantaneous mass inside combustion chamber separately. It was clear that the mass percentage, with reductions in size factor, dropped dramatically during the combustion and expansion process in the same period. When the size factor varied from 1.0 to 0.44, the trapped mass varied from 98.4% to 81.7% for the 5 μm seal gap, and from 95.4% to 26% for the 10 μm seal gap. When the seal gap varied from 5 μm to 10 μm, the trapped mass for size factor 1.0 only decreased by 3.1%, but the trapped mass for size factor 0.44 decreased by 45.7%. Thus, with seal gap increases, the mass loss dramatically increased for the small size factor, but the effect was less pronounced for large size factors.

### 4.4. The Effect of Mass Leakage on the Compression Ratio

**Figure 8:**
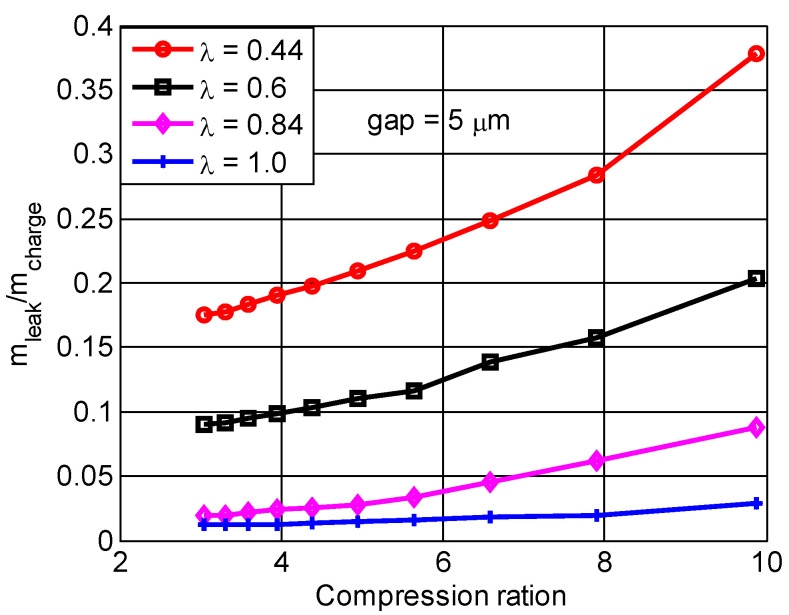
The effect of mass leakage on the compression ratio for different size factors.

Figure 8 shows how the compression ratio affected the nondimensional mass leakage of the combustion chamber when the seal gap was 5 μm, where $m_{\mathrm{charge}}$ and $m_{\mathrm{leak}}$ are the charge and the mass leakage of combustion chamber, separately. Apparently, when the size factor was 1.0, the mass leakage was almost constant with the compression ratio increase. However, when the compression ratio varied from 3 to 5, the mass leakage only increased by 2.2% and 1.8% for size factors 0.4 and 0.6, respectively. When the compression ratio varied from 5 to 9.9, the mass leakage obviously increased by 17.1% and 9.4% for size factors 0.44 and 0.6, respectively. Therefore, it was noted that the mass was only slightly affected when the compression ratio was less than 5. Conversely, the mass leakage increased rapidly with the compression ratio increase. This occurred primarily because the pressure of the combustion chamber and the velocity of mixed gas in the sealed volume increased with the compression ratio increase. It was found that the mass leakage increased obviously with each increase in compression ratio or reduction of size factor.

### 4.5. The Effect of Mass Leakage on the Size Factor

**Figure 9:**
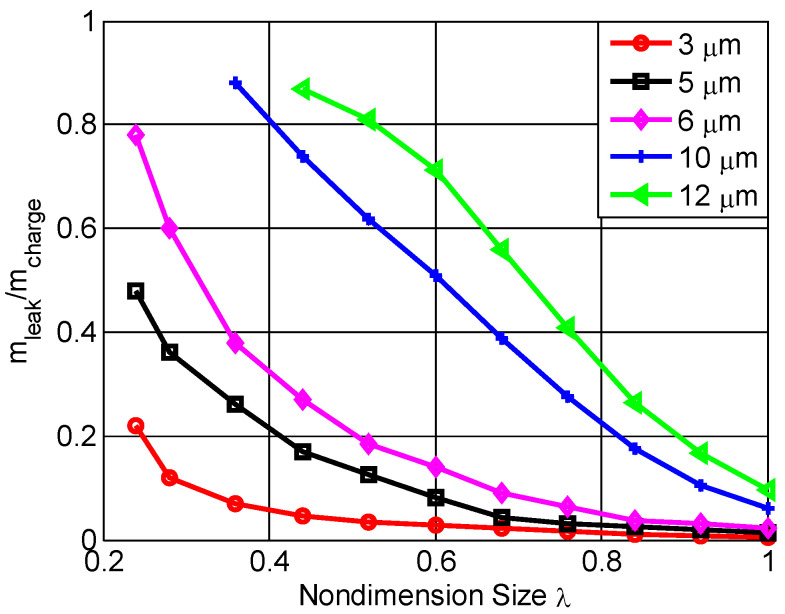
The mass leakage effect on the size factor for different seal gap heights.

Figure 9 shows how the size factors affected the nondimensional mass leakage of the combustion chamber for different seal gap heights. It was observed that the mass leakage rose with increases in seal gap or reductions of size factor. When the seal gap varied from 3 μm to 5 μm and the size factor was more than 0.7, the mass leakage was very low and almost constant. This result was consistent with reference [18], who noted that the 5 μm seal gap had good seal performance. However, for the 3 μm seal gap, the mass leakage was almost invariant when the size factor was more than 0.4; conversely, the leakage increased dramatically. With the size factor reduction, the leakage increased almost linearly, up to more than 80% when the seal gap was more than 10 μm or the size factor was less than 0.4. In practice, the two-stroke MFPSE is unable to work normally with small size factors or large seal gaps. Therefore, for the seal design of a microengine, seal gap height and size factor must be taken into account.

### 4.6. The Effect of Mass Leakage on the Dynamic Viscosity Coefficient

**Figure 10:**
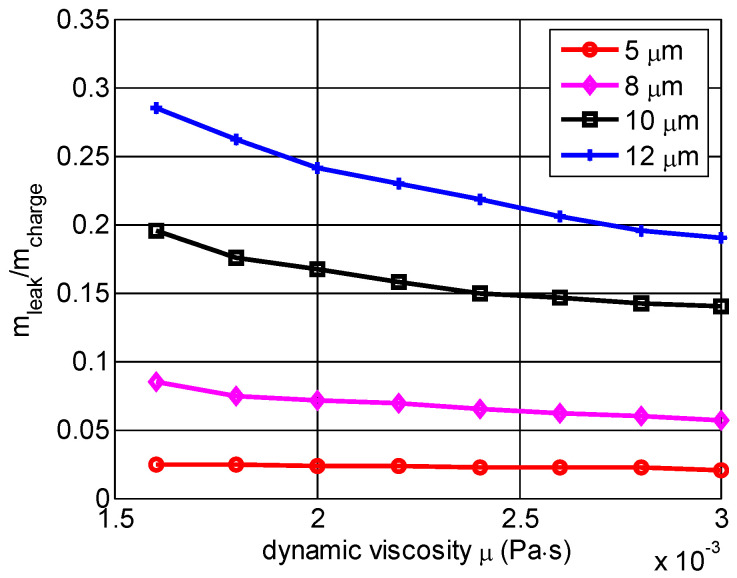
The mass leakage effect on dynamic viscosity coefficient for different seal gap heights.

Figure 10 shows how the dynamic viscosity coefficient affected the nondimensional mass leakage of the combustion chamber for different seal gap heights. The mass leakage was affected only slightly with increases of the dynamic viscosity coefficient when the seal gaps were 5 μm and 8 μm, respectively. For the 10 μm and 12 μm seal gaps, the mass leakage slowly declined with increases of the dynamic viscosity coefficient. We noted clearly that the mass leakage was not significantly affected by changing the dynamic viscosity coefficient for the micro free piston swing engine.

## 5. Conclusions

In this study, a mass leakage model was established for the two-stroke MFPSE. We investigated how different seal gap heights and size factors of micro engines affected the pressure, temperature, and mass leakage. Results were obtained, as follows.

(1) The leakage model of gap seal flow between the body and the center swing was established by the compressible flow Reynolds Navier–Stokes equation.
(2) The nondimensional mass leakage of the combustion chamber was obtained. It was nonlinearly related to the seal gap, size factor, compression ratio, and seal inlet pressure.
(3) When the seal gap was less than 5 μm, the maximum pressure, maximum temperature, and cycle power of combustion chambers were less affected. On the contrary, the maximum pressure and temperature decreased notably with decreases of size factor.
(4) The mass leakage dramatically increased when the seal gap height H>10 μm, the size factor λ<0.4, or the compression ratio $\gamma_{V}>5$. Conversely, the mass leakage was not strongly affected.

This study revealed the various factors that affect mass leakage in a two-stroke micro free piston swing engine, and thus, provided a guide for seal design.

## Figures and Tables

**Table 1:** Parameters of MFPSE.

Initial Parameters	Value
$d_{B}$/mm	46
$d_{H}$/mm	25
$\theta_{B}$/°	120
$\theta_{S}$/°	44
Intake pressure/MPa	0.1
Intake temperature/K	300
Exhaust pressure/MPa	0.1
Exhaust temperature/K	300
Equivalent ratio	0.6

## Data Availability

Not applicable.
